# Smartphone-Delivered Attentional Bias Modification Training for Mental Health: Systematic Review and Meta-Analysis

**DOI:** 10.2196/56326

**Published:** 2024-09-02

**Authors:** Bilikis Banire, Matt Orr, Hailey Burns, Youna McGowan, Rita Orji, Sandra Meier

**Affiliations:** 1 Department of Psychiatry, Faculty of Medicine Dalhousie University Halifax, NS Canada; 2 Faculty of Computer Science Dalhousie University Halifax, NS Canada; 3 Department of Psychology, Faculty of Pure and Applied Sciences Acadia University Wolfville, NS Canada; 4 Faculty of Medicine Dalhousie University Saint John, NB Canada

**Keywords:** attentional bias, mental health problem, anxiety, depression, systematic review, meta-analysis, smartphone, mobile phone

## Abstract

**Background:**

Smartphone-delivered attentional bias modification training (ABMT) intervention has gained popularity as a remote solution for alleviating symptoms of mental health problems. However, the existing literature presents mixed results indicating both significant and insignificant effects of smartphone-delivered interventions.

**Objective:**

This systematic review and meta-analysis aims to assess the impact of smartphone-delivered ABMT on attentional bias and symptoms of mental health problems. Specifically, we examined different design approaches and methods of administration, focusing on common mental health issues, such as anxiety and depression, and design elements, including gamification and stimulus types.

**Methods:**

Our search spanned from 2014 to 2023 and encompassed 4 major databases: MEDLINE, PsycINFO, PubMed, and Scopus. Study selection, data extraction, and critical appraisal were performed independently by 3 authors using the PRISMA (Preferred Reporting Items for Systematic Reviews and Meta-Analyses) guidelines. When necessary, we pooled the standardized mean difference with a 95% CI. In addition, we conducted sensitivity, subgroup, and meta-regression analyses to explore moderator variables of active and placebo ABMT interventions on reducing symptoms of mental health problems and attentional bias.

**Results:**

Our review included 12 papers, involving a total of 24,503 participants, and we were able to conduct a meta-analysis on 20 different study samples from 11 papers. Active ABMT exhibited an effect size (Hedges *g*) of –0.18 (*P*=.03) in reducing symptoms of mental health problems, while the overall effect remained significant. Similarly, placebo ABMT showed an effect size of –0.38 (*P*=.008) in reducing symptoms of mental health problems. In addition, active ABMT (Hedges *g* –0.17; *P*=.004) had significant effects on reducing attentional bias, while placebo ABMT did not significantly alter attentional bias (Hedges *g* –0.04; *P*=.66).

**Conclusions:**

Our understanding of smartphone-delivered ABMT’s potential highlights the value of both active and placebo interventions in mental health care. The insights from the moderator analysis also showed that tailoring smartphone-delivered ABMT interventions to specific threat stimuli and considering exposure duration are crucial for optimizing their efficacy. This research underscores the need for personalized approaches in ABMT to effectively reduce attentional bias and symptoms of mental health problems.

**Trial Registration:**

PROSPERO CRD42023460749; https://www.crd.york.ac.uk/prospero/display_record.php?RecordID=460749

## Introduction

### Background

Smartphone-delivered attentional bias modification training (ABMT) has emerged as a promising intervention for alleviating symptoms of mental health conditions amid a notable increase in their prevalence [1]. As mental health problems, such as anxiety, depression, and substance use disorders, continue to rise globally, traditional treatment options face challenges of accessibility and scalability [1,2]. In response to this growing concern, researchers are exploring innovative approaches such as ABMT, leveraging the ubiquity of smartphones to provide convenient and flexible support for individuals experiencing psychological distress. This systematic review and meta-analysis aimed to evaluate the efficacy of smartphone-delivered ABMT in addressing attentional biases and symptoms of mental health problems, with a particular focus on exploring the impact of different design approaches and methods of administration.

Statistical records have supported a substantial rise, with the number of people with mental health problems increasing from 80.8 million to 125.3 million between 1990 and 2019 [3]. This upward trend has prompted a growing inclination among individuals to seek in-person treatment options for addressing their mental health problems. However, face-to-face therapy also presents societal challenges, including heightened demands on health care systems, a pressing need for additional mental health professionals, and the potential for disparities in access to care [4-6]. Furthermore, various forms of stigma emanate from diverse sources, including families and friends [7]. People’s increasing recognition of the significance of addressing mental health problems explains the urgent requirement for comprehensive and accessible mental health services to effectively tackle the broader societal implications of these conditions, alongside the need to protect individuals’ privacy when seeking mental health assistance.

In addressing the rise in mental health problems, researchers have come up with evidence-based treatments such as pharmacotherapy and psychological interventions that involve medications and behavior modification, respectively [8]. For example, cognitive behavioral therapy (CBT) focuses on modifying behaviors and maladaptive thoughts through language and communication to address dysfunctional cognitions, fostering behavioral change and proving highly effective and versatile across various mental health conditions. Despite the effectiveness of CBT in addressing mental health problems among young individuals, approximately 40% do not exhibit a positive response to this intervention [9,10]. One of the key possible factors of ineffective CBT is the limitation of the youth’s language and communication skills [8]. Therefore, there is continued interest in developing novel interventions.

As options for mental health treatment continue to develop, traditional modalities, such as cognitive restructuring and behavioral activation [11], along with newer approaches, such as third-wave acceptance and mindfulness [12] and ABMT, have been widely used. Prioritizing attentional bias is crucial because it is an automatic process [13]. As such, ABMT can not only be effective in itself but also enhance the effectiveness of other therapeutic interventions and provide a targeted, evidence-based strategy for improving mental health outcomes [14-16].

ABMT stands out as a promising alternative, targeting cognitive processes using visual cues, such as directing attention away from threat-related or addiction-related stimuli. This stimuli-design approach allows ABMT to be more accessible and effective for individuals with limited language and communication skills, overcoming challenges posed by linguistic barriers in the CBT interventions [14,17,18]. Unlike traditional therapies such as CBT, which often involve interpreting complex sentences and verbal interactions, ABMT uses visual and cognitive tasks. For instance, patients respond to visual stimuli rather than needing to interpret text or verbal instructions. This approach reduces the cognitive load and makes it easier for patients to engage effectively in therapy sessions, regardless of their language proficiency or communication abilities. Research indicates that modifying attentional biases through ABMT can have long-lasting effects on emotional regulation and anxiety reduction [19]. ABMT can also be a fully automated, computer-based intervention designed to modify attentional preferences, making it highly scalable and easily accessible for clinical use [16]. In addition, ABMT does not require language communication, which can be particularly advantageous in treating patients who have language barriers or communication impairments.

Recent years have borne witness to a growing interest in ABMT as an empirically supported treatment strategy for an array of mental health problems, including anxiety, posttraumatic stress disorder (PTSD), depression, and substance use. ABMT revolves around the fundamental tenet of training attention away from threat-related stimuli for anxiety, depression, and PTSD and from addictive-cue stimuli for substance use, thereby fostering an internal competition between stimuli that evoke threats or cravings, respectively, and those that are neutral. This internal contest induces a recalibration of attentional mechanisms, leading to a diminished bias toward threat stimuli. The common application of ABMT, grounded in phenomenological characteristics, involves 4 primary experimental tasks: Posner task, Stroop task, dot-probe task, and visual search task [20].

In the context of psychological research, ABMT involves 2 key paradigms: active and placebo ABMT. Active ABMT strategically redirects attention by consistently guiding individuals to focus on neutral stimuli, thereby modifying attentional biases and reducing symptoms associated with anxiety and other mental health issues. In contrast, placebo ABMT serves as a control condition, maintaining the same task structure as active ABMT but placing the cue on both neutral and negative stimuli. This distinction allows researchers to assess the specific therapeutic effects of actively pacing cues to neutral stimuli in ABMT interventions while controlling for nonspecific factors, such as task engagement or participant expectations. These 2 paradigms are pivotal in evaluating the effectiveness of computer-based ABMT and understanding its potential clinical applications [21,22].

The medium through which ABMT is administered has experienced a transformative evolution, aligning itself with the digital tapestry of contemporary health care. While traditionally executed through computer-based platforms, ABMT has recently embarked on a trajectory toward smartphone-mediated delivery [23,24]. This paradigm shift holds great promise, poised to address several pivotal challenges associated with the in-person mode of delivery. The use of smartphones as a conduit for ABMT promises to revolutionize the accessibility and privacy of mental health interventions. The ubiquity of smartphones transcends geographical constraints, rendering mental health support accessible to individuals across diverse locations. In addition, smartphone-based delivery holds the potential to attenuate the omnipresent specter of stigma, an entrenched barrier that has historically dissuaded individuals from engaging with traditional, in-person therapeutic interventions. The discrete and private nature of smartphone-delivered ABMT may sidestep potential stigma, potentially fostering a more expansive adoption of mental health interventions [25]. Furthermore, the incorporation of gamification within smartphone apps enhances user engagement, potentially bolstering treatment adherence and overall efficacy [26]. The gamified interface capitalizes on users’ inherent motivation to participate, cultivates sustained engagement, and optimizes treatment outcomes.

Given the advantages of enhanced accessibility, reduced stigma, increased engagement through gamification, extensive customization, real-time feedback, and seamless integration into daily routines, this review focused on ABMT delivered through smartphones and not ABMT delivered through computers. Recent advancements in psychiatry have seen significant contributions from smartphone-delivered interventions for mental health problems. The previous meta-analyses used a narrow lens for the evaluation of smartphone-delivered ABMT, focusing on specific conditions [27-29]. They found that such interventions were effective in addressing mental health problems, improving quality of life, and reducing symptoms of depression and anxiety. The previous studies focused on smartphone-delivered ABMT on reducing the symptoms of specific mental health problems; however, understanding how ABMT operates across a spectrum of mental health symptoms can help evaluate its effectiveness more comprehensively in reducing symptoms of mental health problems. This review not only assessed the impact of ABMT on a wide range of symptoms of mental health problems but also delved into the mechanisms by which ABMT reduces attentional bias. This investigation included both the active and placebo forms of ABMT. By adopting this comprehensive approach, our review provides insights into how smartphone-delivered ABMT affects not only various mental health symptoms but also attentional bias, offering a more holistic perspective on its effects.

### Objectives

This systematic review and meta-analysis aimed to analyze the efficacy of smartphone-delivered ABMT in reducing mental health symptoms and attentional biases and to understand how different ABMT design strategies influence these outcomes. The mechanisms by which ABMT operates are directly related to these objectives, as ABMT works by retraining the brain to reduce automatic attention to negative stimuli, thus alleviating symptoms of anxiety, depression, and other mental health issues. By modifying attentional patterns through repetitive training tasks, ABMT aims to improve emotional regulation. Different design strategies, such as the type of stimuli, delivery method, and training frequency, may impact the effectiveness of this retraining, making the exploration of these mechanisms essential for optimizing ABMT interventions and achieving better mental health outcomes.

## Methods

### Overview

Our systematic literature review adhered to the Cochrane recommendations [30] and followed the PRISMA (Preferred Reporting Items for Systematic Reviews and Meta-Analyses) guidelines for the planning, execution, and reporting of this study (Multimedia Appendix 1) [31]. In addition, our review protocol was registered with PROSPERO (CRD42023460749).

### Ethical Considerations

In this review, there was no need for informed consent or ethics approval because the data were extracted from previously published studies.

### Search Strategy and Study Selection

In February 2023, MEDLINE, PsycINFO, PubMed, and Scopus were searched systematically for eligible studies published between 2014 and 2023 using keywords related to attentional bias and mobile apps: “attention bias” OR “cognitive bias” AND “smartphone” OR “smartphone application” OR “smartphone app” OR “mobile phones” OR “mobile application” OR “mobile app” OR “personal digital assistant.” These keywords and databases were chosen based on a related prior study [26]. This decision was made to extend their research on the efficacy and design characteristics of ABMT. The rationale behind starting the search in 2023 was based on 2 similar reviews on smartphone-delivered cognitive bias modification interventions, which indicated that the first smartphone-delivered ABMT was developed in 2014 [32,33]. In addition, while other internet-based ABMT interventions can be accessed on mobile phones, our focus is exclusively on interventions developed specifically for mobile phones. Recognized articles were exported to the web-based systematic review software Rayyan (Rayyan Systems, Inc) [34], and duplicates were removed. The remaining articles were reviewed for inclusion by 3 independent authors using Rayyan.

### Inclusion and Exclusion Criteria

**Figure 1:**
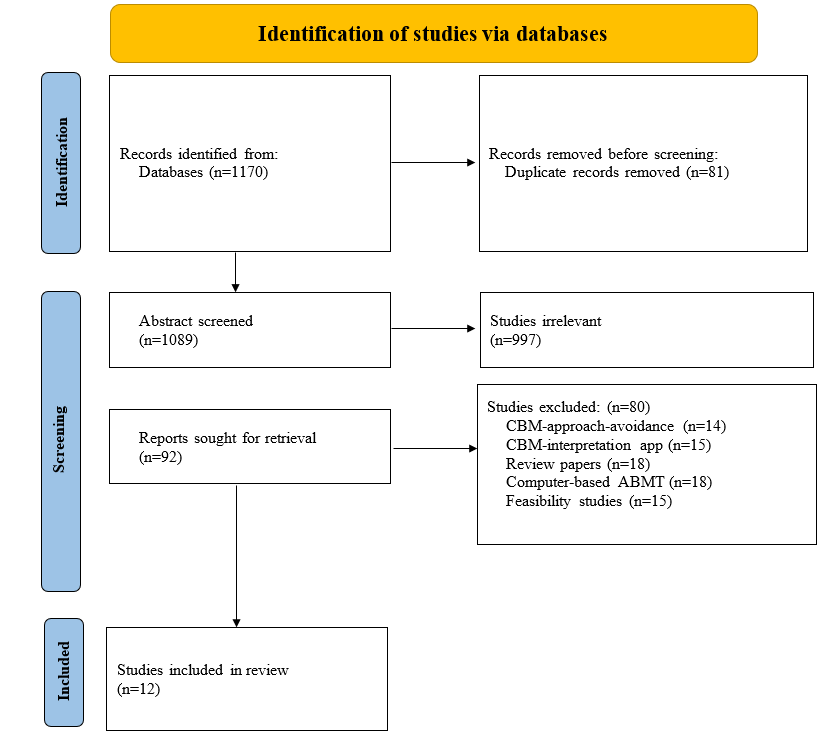
PRISMA (Preferred Reporting Items for Systematic Review and Meta-Analyses) flowchart. ABMT: attentional bias modification training; CBM: cognitive bias modification.

To be considered for inclusion in this review, studies had to satisfy the following criteria: they had to be written in English and assess ABMT for mental health symptoms, such as anxiety, depression, stress, PTSD, or substance use (eg, smoking and alcohol consumption). ABMT interventions were explicitly defined and limited to those delivered exclusively through mobile devices. We included studies that met the following conditions: (1) ABMT was administered using a mobile device, such as a mobile phone, smartphone, or PDA, and (2) the delivery method took the form of a dedicated app or game. Articles that met any of the following criteria were excluded: (1) reviews, (2) interpretation bias modification delivered in any format, (3) avoidance bias modification delivered in any format, (4) web-based ABMT, and (5) computer-based ABMT. Web-based and computer-based ABMT interventions are similar in that both aim to modify attentional biases by training individuals to shift their focus away from negative stimuli. They offer interactive tasks and can be accessed remotely. However, web-based ABMT is accessed through an internet browser, making it more flexible and accessible across various devices, whereas computer-based ABMT usually requires specific software installed on a computer, potentially limiting accessibility. The objective of this review was to investigate ABMT that can be accessed only through smartphones, excluding the option of using other types of devices, to understand its unique effectiveness and accessibility. Therefore, ABMT interventions delivered through other platforms were excluded from this review. Figure 1 provides a visual representation of the inclusion and exclusion processes.

### Data Extraction and Risk-of-Bias Assessment

We used the Rayyan management software to initially screen studies based on their titles and abstracts. Subsequently, 3 authors (BB, MO, and HB) independently performed data extraction based on the predetermined eligibility criteria. The disagreements that arose during this process were effectively resolved through collaborative discussion among the authors. The data extracted from each included study encompassed several key elements: author information, publication date, sample size, the delineation of sample groups (active and placebo), the description of the type of treatment, specifics regarding experimental tasks (dot-probe task, Stroop task, or visual search task), the type of threat stimuli (faces, pictures, or words), characteristics of threat stimuli, details about the number of stimuli and the type of stimulus array presented, the number of trials and sessions, stimulus presentation duration, and the outcome measurements used. Of the 5 authors, 2 (BB and YM) independently used the Cochrane risk-of-bias assessment tool to evaluate the risk of bias in the selected studies for the meta-analyses [35]. We also discussed the discrepancies to reach a consensus.

### Data Analysis

We used the R Studio analysis packages (The R Foundation) [36] to conduct the meta-analyses. To perform these analyses, we used sample sizes for each group (active and placebo), along with means and SDs of mental health symptoms and attentional biases observed before and after the intervention (preintervention and postintervention assessments). These data were instrumental in calculating meta-estimates for both attentional bias levels and the reduction in mental health symptoms, encompassing anxiety, depression, stress, PTSD, or substance use. These meta-estimates were derived through random-effects meta-analyses.

The rationale for choosing random-effects meta-analyses is the anticipated substantial heterogeneity and aim to obtain a comprehensive overview of the true effect size while accounting for the variability among studies. Random-effects meta-analyses, fixed-effects meta-analyses, and Bayesian meta-analyses are common types of meta-analyses, each with distinct characteristics [37]. The choice of a random-effects model is often preferred when conducting a meta-analysis due to several key reasons. First, it is a flexible approach that can accommodate significant heterogeneity, which is common in meta-analyses involving diverse study populations and research questions. By allowing for varying effect sizes between studies, the random-effects model acknowledges the inherent variability in study results and provides more conservative estimates with wider CIs. This conservative approach is valuable, as it acknowledges the uncertainty associated with the underlying effect sizes and is less influenced by potential outliers.

The primary meta-analysis aimed to compute a comprehensive Hedges *g* effect size, accompanied by 95% CIs. This effect size was computed for both active and placebo ABMT interventions across all the studies included in our analysis. To interpret the effect sizes, we applied the Hedges *g* values of 0.20, 0.50, and 0.80, which correspond to small, moderate, and large effect sizes, respectively [38]. Heterogeneity was quantified using the *I*^2^ statistic, and *I*^2^>50% was considered evidence of substantial heterogeneity. We also used the inverse variance approach, a restricted maximum-likelihood estimator for τ^2^, and the Q-profile method to establish CIs for τ^2^ and τ, ensuring a robust analytical framework. Publication bias was examined using funnel plots, and the presence of asymmetry was assessed using the Egger regression test [39]. If the Egger test yields a significant result (indicating asymmetry), it suggests potential publication bias in the meta-analysis.

In addition, we performed separate sensitivity analyses using meta-regression with random-effects models in cases where there were enough study samples (at least 3), drawing reference from a previous study [27]. Sensitivity analysis is crucial for assessing the robustness of meta-analysis results and understanding the impact of potential sources of bias or variability, particularly in the presence of significant heterogeneity [40]. Moderators refer to specific factors or variables that can influence the relationship between the use of the smartphone-delivered ABMT intervention and its impact on mental health symptoms or attentional bias. These moderators can help researchers better understand the conditions under which the smartphone-delivered ABMT is effective and provide insights into the nuances of its outcomes. These meta-regression analyses allowed us to explore the influence of 5 moderators identified from the ABMT design characteristics reviewed, namely type of threat stimuli (face, images, or words); stimulus array type (left right or top down); design style (gamified or not gamified); display duration (200 ms or 500 ms) where applicable, along with the risk of bias (low or some concerns); and treatment groups (mental health and attentional bias) as additional considerations. These factors were identified as potential moderators.

## Results

### Study Selection

The initial search produced 1170 results, and we reduced this number to 1089 (93.08%) by eliminating duplicates. We excluded 997 (91.55%) articles after reviewing their titles and abstracts. Moving on to the full-text level, we reviewed the remaining 92 (8.4%) articles. Out of these 92 articles, we retained only 12 (13%) articles that were relevant to this paper. During the extraction process, we excluded 80 (87%) articles out of the initially considered 92. The article screening process is detailed in Figure 1. In Table 1, we have summarized the characteristics of the included studies. The sample sizes varied, ranging from 18 to 22,993 participants across different studies. The studies evaluated active and placebo interventions for 5 different symptoms identified in 24,503 participants. These 5 symptoms include anxiety, depression, stress, PTSD, and substance use. The treatments involved tasks used include dot-probe, Stroop, and visual search tasks, with threat or addictive-cue stimuli, including faces, words, and images. The duration of stimulus presentation was typically 500 milliseconds, and the number of stimuli varied between 2 and 16 per array type. The number of trials conducted in these studies ranged from 60 to 800. Overall, these studies encompassed a diverse range of sample sizes and characteristics, reflecting their focus on different mental health problems and intervention strategies.

**Table 1:** Study characteristics (N=12).

Study	Sample size, N	Active, n (%)	Placebo, n (%)	Treatment	Tasks	Threat or addictive-cue stimuli	Duration (ms)	Stimulus, n and array type	Trials, N
Dennis and O’Toole [41], 2014	76	Short: 18 (24); long: 19 (25)	Short: 20 (26); long: 19 (25)	Anxiety	Dot probe	Face	500	2 and top down	640
Enock et al [42], 2014	326	158 (48.5)	141 (43.3); WL^a^: 27 (8.3)	Anxiety or depression	Dot probe	Face	500	2 and top down	160
Dennis-Tiwary et al [43], 2016	42	19 (45)	23 (55)	Anxiety	Dot probe	Face	500	2 and top down	480
Yang et al [44], 2017	40	20 (50)	20 (50)	Anxiety	Dot probe	Face	500	2 and top down	800
Dennis-Tiwary et al [24], 2017	29	15 (52)	14 (48)	Anxiety or stress	Dot probe	Face	500	2 and top down	160
Teng et al [45], 2019	82	30 (37)	30 (37); WL: 22 (27)	Anxiety	Dot probe	Word	500	Left and right	82
Flaudias et al [46], 2020	41	18 (44)	Memory group: 15 (37); no AB^b^: 8 (20)	Alcohol use	Stroop	Image	500	3 and grid	240
Niles et al [47], 2020	546	Personalized: 177 (32.4); nonpersonalized: 179 (32.8)	190 (34.8)	Anxiety or PTSD^c^	Dot probe	Word	500	2 and top down	70
Charvet et al [23], 2021	35	High anxiety: 17 (49); low anxiety: 13 (37)	No placebo group	Anxiety or depression	Dot probe	Face	500	2 and top down	120
Chelliah and Robinson [48], 2022	22,993	Dot 4448: 4448 (19.34); Dot 2588: 2588 (11.26)	Dot 4301: 4301 (18.7); Dot 4818: 4818 (20.95); no training: 6778 (29.47)	Anxiety	Visual search	Face	500	16 and grid	100
Flaudias et al [49], 2022	47	20 (43)	27 (57)	Alcohol use	Stroop	Image	500	4 and grid	60
Robinson et al [50], 2022	246	124 (50.4)	122 (49.6)	Anxiety or substance use disorder	Dot probe and Stroop	Image and word	500	2 and left right	440

^a^WL: waiting list.

^b^AB: attentional bias.

^c^PTSD: posttraumatic stress disorder.

### Risk of Bias in the Studies

**Figure 2:**
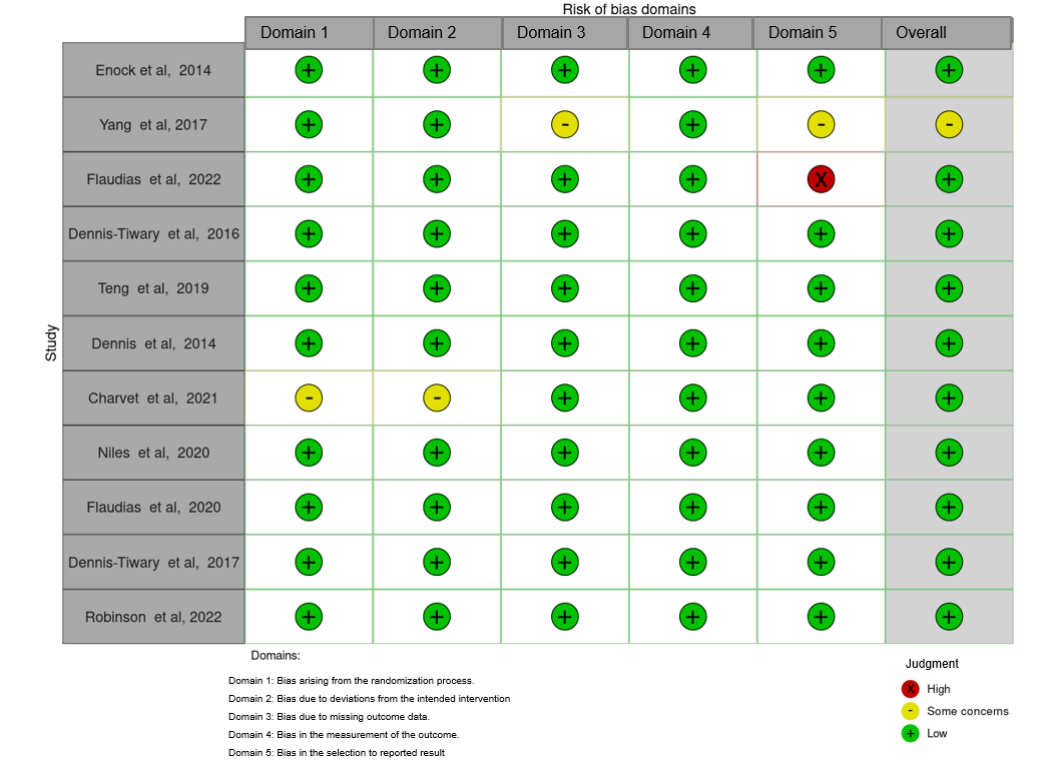
Risk-of-bias domains.

**Figure 3:**
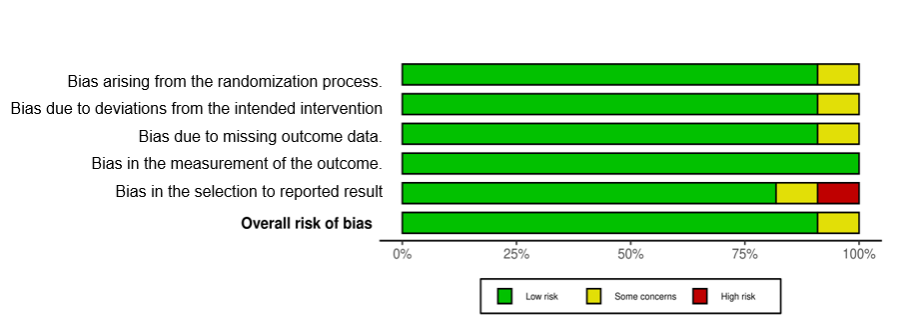
Overall risk of bias.

The risk-of-bias assessment for the included studies was conducted across 5 specific domains (Figure 2 [23,24,41-47,49,50] and Figure 3): *randomization*, *deviation from intended intervention, missing outcome data, measurement of the outcome, and selection of the reported result.* Among the 11 studies analyzed, the majority demonstrated a low risk of bias across all these domains, indicating a generally robust methodology in these investigations. Specifically, 9 (82%) out of 11 studies were categorized as having a low overall risk of bias, implying a high level of confidence in their research findings. However, 1 (9%) out of 11 studies raised some concerns, primarily in the domains of randomization, deviation from intended intervention, and missing outcome data. In addition, 1 (9%) out of 11 studies showed a high risk of bias, particularly in the domain of measurement of the outcome. Our assessment of the risk of bias in individual studies is presented in Multimedia Appendix 2 [23,24,41-47,49,50]. These findings underscore the overall quality and reliability of the studies, with the majority (9/11, 82%) exhibiting a low risk of bias in their design and execution, while a small proportion raised some concerns (1/11, 9%) or demonstrated a high risk of bias (1/11, 9%) in specific domains.

### Effectiveness of Smartphone-Delivered Active ABMT for Mental Health Symptoms

**Figure 4:**
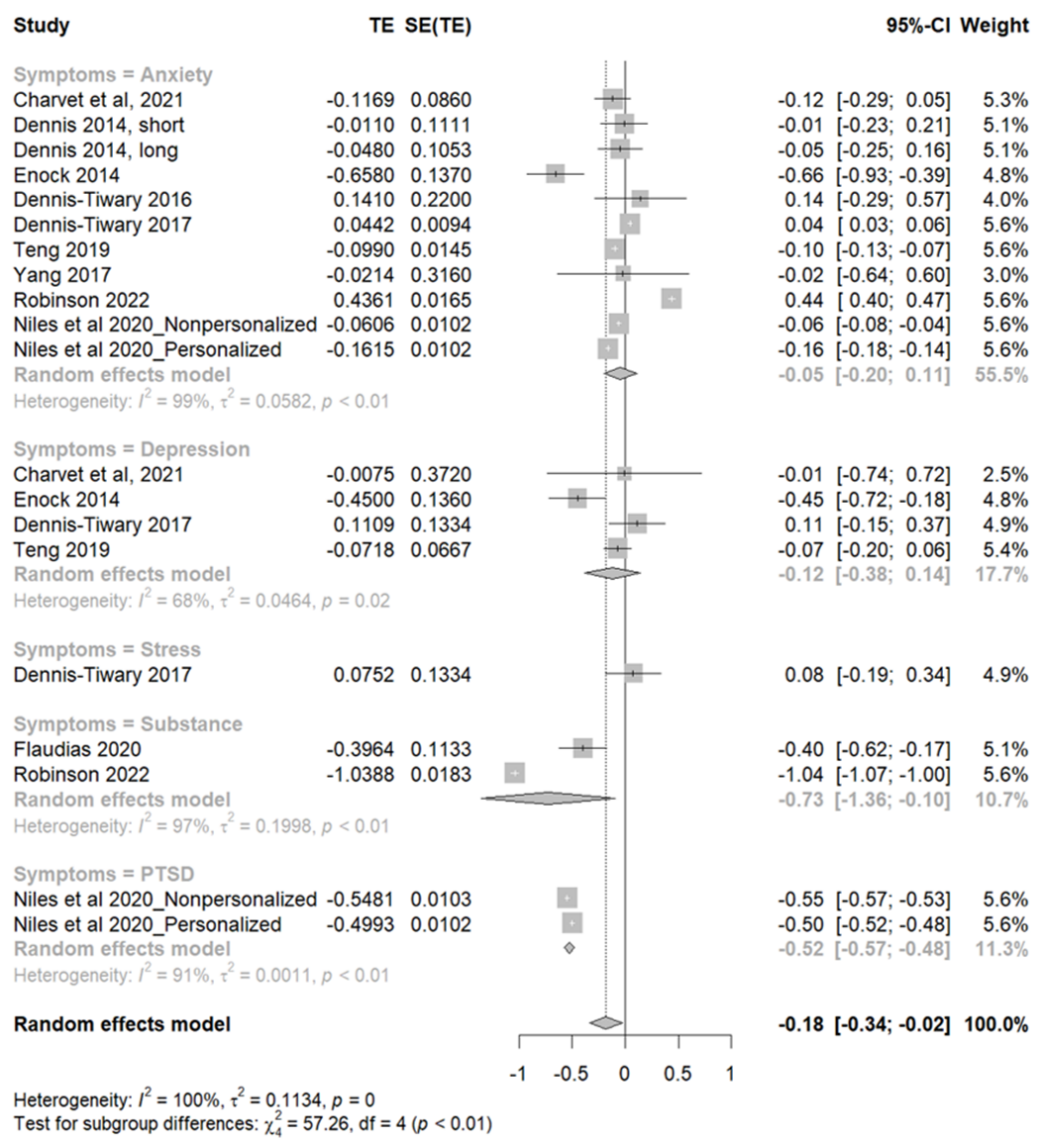
Forest plots for active attentional bias modification training for mental health symptoms. PTSD: posttraumatic stress disorder; TE: treatment effect.

In this comprehensive analysis involving 20 study samples (Figure 4 [23,24,41-45,47,50]), the pooled effect size for the study samples reflected a significant effect of active ABMT on mental health symptoms (Hedges *g*=–0.18, 95% CI –0.340 to –0.02; *z* score=–2.27; *P*=.03). Specifically, the negative value of the effect size suggests that the symptoms decreased after the intervention. In addition, the statistical tests conducted confirm that this reduction is unlikely to have occurred by chance, suggesting that active ABMT can be effective in alleviating mental health symptoms. Significant heterogeneity was observed among the study samples (*Q*=6526.76; *P*<.001; *I*^2^=99.7%). The subgroup meta-analysis revealed diverse effects of interventions across 5 distinct symptom categories, namely anxiety, depression, stress, substance use, and PTSD, with the varying impacts of interventions and heterogeneity. Sensitivity analysis of the compiled *P* values presented in Multimedia Appendix 3 [23,24,41-47,49,50] showed that specific studies, including the studies by Dennis-Tiwary et al [43] (*P*=.02) and Robinson et al [50] (*P*=.005), emerged as influential contributors to the overall significance. Despite their exclusion, the meta-analysis maintained statistical significance, reaffirming the primary findings’ solidity. The test of the asymmetry funnel plots presented in Multimedia Appendix 4 showed no evidence of publication bias (t_18_=0.31, *P*=.76).

### Effectiveness of Smartphone-Delivered Placebo ABMT for Mental Health Symptoms

**Figure 5:**
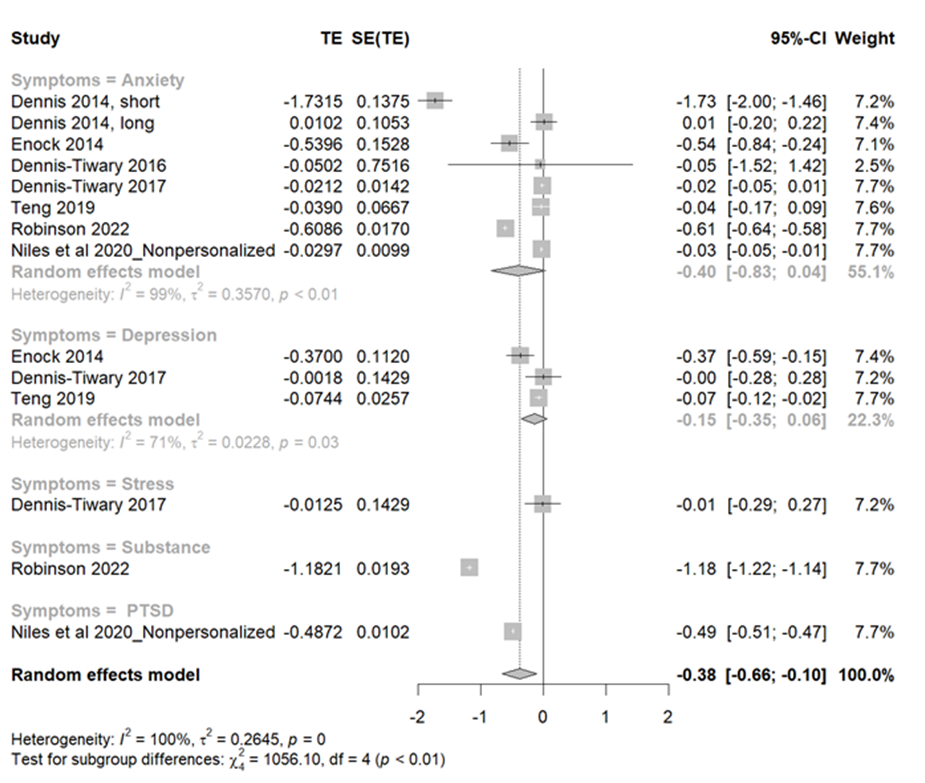
Forest plots for placebo attentional bias modification training for mental health problems. PTSD: posttraumatic stress disorder; TE: treatment effect.

In this analysis involving 14 study samples (Figure 5 [24,41-43,45,47,50]), the outcomes revealed a significant effect size (Hedges *g*=–0.381, 95% CI –0.8307 to 0.0403; *z* score=–2.66; *P*=.008), and significant heterogeneity was observed among the samples (*Q*=559.83; *P*=.002; *I*^2^=99.7%). In essence, the negative value of the effect size suggests that the symptoms decreased after the intervention. In addition, the statistical tests conducted confirm that this reduction is unlikely to have occurred by chance, suggesting that placebo ABMT can be effective in reducing mental health symptoms. In the subgroup analysis, diverse effects of interventions across 5 distinct symptom categories, namely anxiety, depression, stress, substance use, and PTSD, were observed. Sensitivity analysis of the compiled *P* values presented in Multimedia Appendix 3 revealed that among the 14 study samples considered, the exclusion of 2 specific study samples, short active AMBT (*P*=.009) and long active AMBT (*P*=.006) from the study by Dennis and O’Toole [41], was found to exert substantial influence, significantly impacting the overall statistical significance. Notably, even with the removal of these influential study samples, the overall analysis sustained its statistical significance. The results of the test of the asymmetry funnel plots are displayed in Multimedia Appendix 4. The results of the linear regression test conducted to assess funnel plot asymmetry yielded a nonsignificant outcome (t_12_=–0.35, *P*=.73), indicating that there was no publication bias.

### Effectiveness of Smartphone-Delivered Active ABMT for Attentional Bias

**Figure 6:**
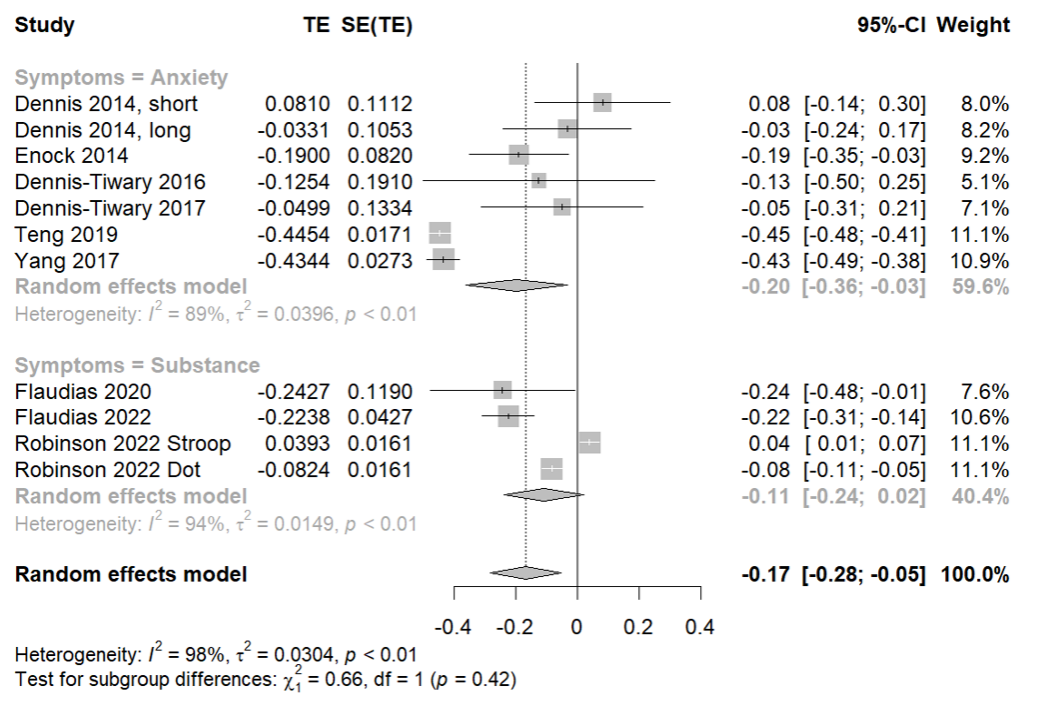
Forest plots for active attentional bias modification training for attentional bias.

In the analysis involving 11 study samples evaluating attentional bias, we observed a significant outcome using a random-effects model (Figure 6 [24,41-45]). The pooled effect size analysis for the study samples showed a significant effect of active ABMT on attentional biases (Hedges *g*=–0.17, 95% CI –0.28 to 0.05; *z* score=–2.87; *P*=.004). In summary, the negative value of the effect size suggests that the attentional bias decreased after the intervention, and the statistically significant effect did not occur by chance, suggesting that active ABMT is effective in reducing attentional bias.

Significant heterogeneity was observed among all the samples (*Q*=559.83; *P*=.004; *I*^2^=98.2%). The analysis of the subgroups within the study samples showed different effects and significant heterogeneity among the 2 different category symptoms: anxiety and substance use. Sensitivity analysis of the compiled *P* values presented in Multimedia Appendix 3 revealed that even after the removal of specific study samples, the overall analysis maintained statistical significance, reaffirming the efficacy of ABMT in reducing attentional bias. The test of the asymmetry funnel plot is displayed in Multimedia Appendix 4. The Egger regression test found no evidence of publication (*t*_9_=–0.09, *P*=.93).

### Effectiveness of Smartphone-Delivered Placebo ABMT for Attentional Bias

**Figure 7:**
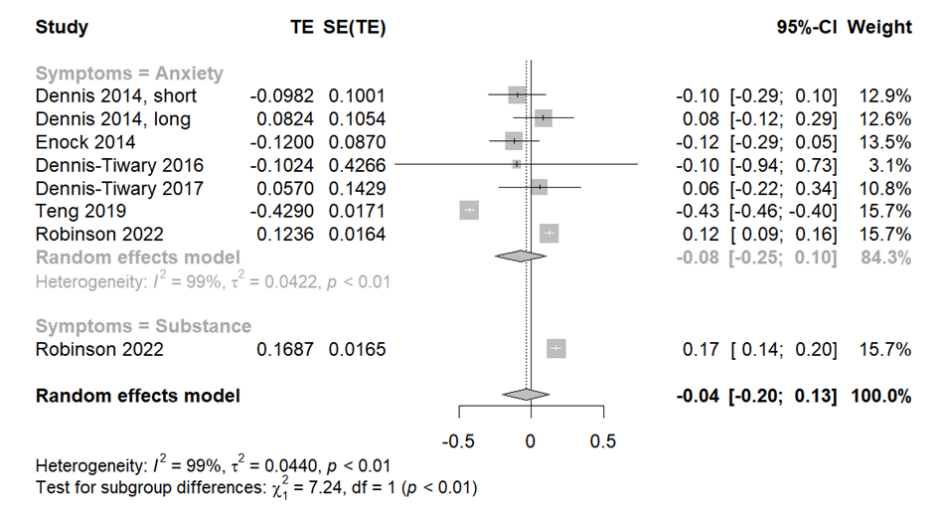
Forest plots for placebo attentional bias modification training for attentional bias.

In this analysis comprising 8 study samples, as shown in Figure 7 [24,41-43,45,50], the pooled effect size did not reflect a significant effect of placebo ABMT on attentional biases (Hedges *g*=–0.04, 95% CI 0.200-0.13; *z* score=–.44; *P*=.66). The negative value of the effect size suggests that the attentional bias decreased after the intervention; however, the effect size was not statistically significant, suggesting that placebo ABMT is not effective in reducing attentional bias. Significant heterogeneity was observed among the study samples (*Q*=779.84; *P*<.001; *I*^2^=99.1%). The subgroups within the random-effects model unveiled varying effects and heterogeneity linked to the different symptom categories of anxiety and substance use. The sensitivity analysis based on the compiled *P* values in Multimedia Appendix 3 indicated that even when certain studies were excluded, the overall analysis did not attain statistical significance. The test of asymmetry funnel plot is displayed in Multimedia Appendix 4; however, the analysis was not conducted because the study samples were too small to be included in the meta-regression, but the funnel plot showed asymmetry.

### Moderator Analyses

The moderator analysis focused exclusively on anxiety and depression, given the limited samples in other subgroups. Only the anxiety subgroup is discussed, as the depression subgroup did not exhibit significant effects on all the moderating parameters, as shown in Multimedia Appendix 5 based on the raw data provided in Multimedia Appendix 6 [23,24,41-47,49,50]. Results of the moderator analysis using meta-regression showed that the choice of “stimuli” played a significant role in shaping treatment outcomes. Specifically, when “images” were used as stimuli for individuals with anxiety symptoms, a significant effect on anxiety reduction was observed, suggesting that this stimulus type may be particularly effective in this subgroup. In contrast, the use of “face” stimuli for patients with primary anxiety symptoms and “word” stimuli for those with anxiety and PTSD did not yield significant effects on reducing anxiety symptoms, and the use of “word” stimuli for those with anxiety related to PTSD did not yield significant effects on anxiety outcomes.

Moreover, the “display duration” of 200 milliseconds emerged as a significant moderator (β=.436; *P*=.002), indicating that shorter exposure durations may lead to more substantial reductions in symptoms, while a longer duration of 500 milliseconds showed a negative effect on outcomes (β=–.537; *P*<.001). The other factors, such as “stimulus array type,” “design style,” and “risk of bias,” did not exhibit significant moderating effects on anxiety outcomes within ABMT interventions. These findings illustrate the importance of tailoring ABMT interventions based on the specific type of threat stimuli and the characteristics of the target population to optimize their efficacy, thus highlighting the nature of ABMT’s impact on anxiety reduction.

## Discussion

### Principal Findings

#### Overview

This paper presents a systematic review and meta-analysis of 12 individual studies including 20 independent samples. The overarching goals were to (1) compute the overall effect sizes for active ABMT and placebo ABMT on the reduction of attentional bias and symptoms of mental health problems and (2) separately evaluate which ABMT design characteristics moderate the effect sizes in reducing mental health problems and attentional bias. Importantly, this analysis included only randomized controlled trial designs and study samples that included pre-post modification comparisons of mental health symptoms and attentional biases. Our study diverges from earlier reviews focused on gamification elements and commercialized apps by prioritizing the core phenomenological characteristics of ABMT: Posner task, Stroop task, dot-probe task, and visual search task. Embedding these established tasks in smartphone interventions ensures scientific rigor and enhances ABMT effectiveness. Furthermore, our research identifies current smartphone approaches and explores novel adaptations of these tasks for optimal integration into mobile platforms. This exploration provides practical insights for designers and developers, guiding interface design, interaction mechanics, and task presentation to boost engagement and adherence among mobile users. These insights are pivotal for advancing mobile-based ABMT, ensuring interventions are scientifically grounded and efficacious in enhancing mental health outcomes. In addition, our study represents the first meta-analysis focused on smartphone-based ABMT. Unlike previous reviews, we included both placebo and active designs to rigorously assess the efficacy of smartphone-based ABMT interventions. Using a randomized controlled trial design with a diverse participant sample, we enhanced methodological robustness and applicability. These contributions set a high standard for future research in smartphone-based ABMT, aiming to improve the accessibility and effectiveness of mental health interventions broadly.

#### Overall Effect

Overall, this review found that using ABMT through smartphones had a small but significant impact (Hedges *g*=0.17), whereas Hakamata et al [51] reported a larger effect size (Cohen *d*=0.51). The studies by Heeren et al [52] and Hang et al [53] reported small effect sizes for computer-based ABMT in reducing anxiety, with Hedges *g*=0.41 for social anxiety and Hedges *g*=0.26 for anxiety disorders, respectively. It is important to note that the study by Hakamata et al [51] encompassed various delivery methods without a specific focus, while the studies by Heeren et al [52] and Hang et al [53] concentrated on the computer-based medium. In contrast, this review’s investigation specifically centered on ABMT via smartphones. These results highlight that both smartphone-based and computer-based ABMT interventions can be effective in reducing social anxiety symptoms, despite variations in effect size. The effect size variation across media may be due to the difference in screen sizes between computers and smartphones. Exploring the differences in platforms for the delivery of ABMT will be insightful intervention for mental health problems . Despite variations in effect size between studies, the findings by Hakamata et al [51] reinforce the notion that design characteristics play a crucial role in determining the effectiveness of ABMT interventions, aligning with the observations of this study. This suggests that while the magnitude of the effect may differ, the underlying factors contributing to the efficacy of ABMT remain consistent across different research contexts. In essence, the emphasis on design characteristics highlights the need for the careful consideration of intervention parameters to optimize outcomes in ABMT research and practice. Furthermore, the meta-analysis of combined ABMT and CBT also showed small but significant effects on clinician-rated anxiety symptoms and attentional bias toward threat [54]. Smartphone-delivered ABMT showed similar effects to other mental health apps for anxiety and depression [27,55,56]. In contrast, the placebo ABMT showed a small and not significant effect size for reducing attentional bias toward negative stimuli (Hedges *g*=–0.04; *P=*.66), but there was a significant small to moderate effect size for reducing mental health for anxiety and depression (Hedges *g*=–0.38; *P=*.008).

Our findings on placebo ABMT revealed a significant and moderate effect size in reducing mental health problems but not attentional bias. Other studies have shown that both active and placebo ABMT interventions significantly reduce mental health problems [21,45,57], which supports our findings. These findings regarding symptom reduction across active and placebo ABMT interventions suggest a potential placebo effect, emphasizing the need for further investigation into the mechanisms driving these outcomes and the broader implications for understanding placebo treatment effects in therapeutic interventions.

The significant effects of both active ABMT and placebo ABMT on symptoms of mental health problems can be explained through several mechanisms supported by previous studies. Active ABMT works by specifically targeting and modifying attentional biases toward threat-related stimuli, which are often implicated in anxiety and other mental health conditions. This modification reduces cognitive load and emotional reactivity, leading to symptom reduction [58,59]. In addition, active ABMT enhances emotional regulation by training individuals to redirect their attention away from negative stimuli. In contrast, placebo ABMT may improve symptoms through expectation effects, where participants’ belief in receiving effective treatment triggers neurobiological responses contributing to symptom improvement [60]. Moreover, the structured nature of placebo ABMT improves therapeutic engagement and support, enhancing feelings of control and self-efficacy [42]. Finally, the cognitive engagement involved in placebo ABMT tasks can improve cognitive functioning, indirectly benefiting mental health. These mechanisms collectively explain the significant effects observed in both treatment conditions.

The significant heterogeneity observed in the effectiveness of smartphone-delivered active ABMT for mental health symptoms across studies could be attributed to several factors. First, there are variations in study populations and sample sizes, ranging from small groups of 29 participants [43] to large samples of 22,993 participants [48], which could affect the generalizability and robustness of the results. Second, the type of mental health conditions addressed varies, with studies targeting anxiety, depression, PTSD, alcohol use, and substance use disorders, potentially leading to different outcomes based on the specific symptoms being treated. Third, the intervention protocols differ significantly; for instance, while most studies use the dot-probe task with face stimuli, others use the Stroop task with images or words or a visual search task. In addition, the duration of exposure to stimuli and the number of trials vary widely, from 60 trials [49] to 800 trials [44], which might influence the effectiveness of the intervention. These methodological differences, including those in the type of stimuli, the structure of the tasks, and the specific parameters of the intervention, contribute to the observed heterogeneity in the outcomes of these studies.

However, sensitivity analysis emerged as a critical tool in navigating this heterogeneity, allowing for a thorough examination of the robustness of the findings [27]. Despite the significant observed heterogeneity among the study samples, sensitivity analysis revealed that the meta-analysis maintained its statistical significance even after excluding influential studies. This indicates that the overall findings remained robust and reliable, despite the presence of variability across the included studies. By systematically assessing the impact of individual studies or study characteristics on the overall results, sensitivity analysis provided valuable insights into the stability of the meta-analysis outcomes.

#### Comparison of Smartphone-Delivered ABMT With Computer-Delivered ABMT

The current findings on smartphone-delivered ABMT and its effects on attentional bias and mental health symptoms, particularly for anxiety and depression, are compared with mixed significant effects reported in other meta-analyses on ABMT delivered through other platforms, that is, computer-based (internet-based) ABMT interventions.

Considering computer-based ABMT platforms, the study by Heeren et al [52] primarily focused on their application for social anxiety disorder. The meta-analysis reported small but significant effect sizes for ABMT in reducing attentional bias (Hedges *g*=0.30) and social anxiety disorder symptoms (Hedges *g*=0.41 for training toward neutral stimuli vs control condition) after multiple sessions. The study also noted that the control conditions often performed similarly to the ABMT, suggesting a potential placebo effect or the nonspecific benefits of participating in a study. Similarly, the study by Hang et al [53] focused on computer-based ABMT but extended the discussion to children and adolescents with anxiety disorders. This meta-analysis found that ABMT had small but significant effects on clinician-rated anxiety symptoms (Hedges *g*=0.26) and attentional bias toward threat (Hedges *g*=0.21) but not on self-reported or parent-reported anxiety measures (Hedges *g*=–0.08). The control conditions used in these studies, such as attention control training, did not show significant effects, which contrasts with the significant effects seen in this study for placebo ABMT on reducing mental health symptoms for anxiety and depression.

This review’s findings suggest a potential advancement in the delivery method of ABMT. The use of smartphones could increase accessibility and adherence to ABMT protocols, potentially enhancing their effectiveness. These results build upon the findings from the studies by Heeren et al [52] and Hang et al [53] by suggesting that the delivery method of ABMT (eg, via smartphone) and the nature of the control condition can significantly influence the outcomes of ABMT interventions. They also highlight the importance of considering placebo effects in the design and interpretation of ABMT studies, as nonspecific factors can sometimes produce significant improvements in mental health symptoms. This underscores the need for well-designed studies to carefully assess the specific contributions of ABMT techniques to changes in attentional bias and mental health outcomes.

#### Moderating Factors of ABMT Intervention

The moderator analysis conducted in this review comprehensively evaluated the effectiveness of ABMT design characteristics across various mental health problems, including anxiety, depression, PTSD, and substance use disorders. The analysis shows that certain characteristics, such as the threat stimulus type, spatial arrangement of stimuli, and duration of stimuli display, have an influence on ABMT’s effectiveness. This finding is consistent with a previous meta-analysis that showed that the use of ABMT interventions may be owing to their varying design characteristics and optimal protocols (eg, task types, target stimuli, stimulus directions, and display settings) [61]. However, due to the limited number of study samples (<3) available in the other mental health problem categories, the analysis focused only on anxiety and depression. Delving into the intricacies of ABMT, we first explored the role of its design characteristics, including the type of threat stimuli, the spatial arrangement of stimuli, and the duration of stimuli display, in influencing ABMT’s effectiveness.

Given the findings from our moderator analyses highlighting the significant influence of various design characteristics on ABMT’s effectiveness, there is a compelling rationale for exploring personalized approaches in future smartphone-based ABMT interventions. While our review did not directly analyze personalized ABMT due to the limited studies with personalized features, the identified moderating factors offer valuable insights into potential avenues for customization. For example, the choice of stimuli, spatial arrangement, and the duration of stimulus display emerged as critical factors influencing treatment outcomes. Building on these insights, future research could investigate how tailoring ABMT protocols to individual preferences and needs, based on these design characteristics, could enhance treatment efficacy. By developing personalized ABMT interventions that align with patients’ specific attentional biases and cognitive profiles, we may optimize treatment outcomes and improve overall engagement and adherence. Therefore, we propose personalized approaches as a promising direction for further exploration in the field of smartphone-delivered ABMT interventions.

#### Personalization of ABMT Interventions on Smartphone Platforms

Personalization in the context of ABMT refers to the customization of intervention components to align with individual preferences, needs, and characteristics. This customization can encompass various aspects of the intervention, including the stimulus selection, presentation format, difficulty levels, and session duration. By tailoring the intervention to everyone, personalization aims to enhance engagement, relevance, and effectiveness, ultimately optimizing treatment outcomes.

The personalization of ABMT interventions on smartphone platforms and that of ABMT interventions on PCs can differ significantly due to the unique characteristics and capabilities of each device. However, the personalization of ABMT design can be achieved on both smartphones and PCs by adding the option of selecting the type of stimuli and duration of stimuli display. However, personalization on smartphone platforms tends to be more dynamic, context aware, and automated, leveraging the device’s sensors and data processing capabilities to tailor ABMT interventions to the user’s current context and needs.

Smartphone-based ABMT offers unique advantages for personalization compared to traditionally delivered (computer-based) ABMT. With smartphones, users have greater control and flexibility in customizing intervention parameters according to their preferences. For example, individuals with anxiety may respond differently to various types of stimuli, as highlighted by this study. Smartphone apps can allow users to select their preferred stimulus types, such as images, faces, or words, based on their personal preferences and comfort levels. This level of customization enables users to engage with stimuli that resonate the most with them, potentially enhancing their attentional training experience and improving treatment outcomes.

Similarly, smartphones can be leveraged to track personalized data, such as location and physical activity patterns, using their sensors. These data may be used to inform real-time feedback on user performance. In contrast, personalization on PCs may rely more on user input and manual customization, offering greater control but potentially limiting the adaptability and responsiveness of the intervention. The use of mobile sensors in personalizing mobile health interventions is highlighted in the meta-analysis by Tong et al [62]. The study found that interventions using system-captured data, which can be obtained from mobile sensors, were associated with higher effectiveness compared to those using user-reported data. Specifically, the standardized difference in means for interventions using system-captured data was 1.48 (95% CI 0.76-2.19), indicating a more substantial impact on lifestyle behavior outcomes when mobile sensors are used for personalization. Despite the advantages of the smartphone-delivery platform, it also comes with potential challenges, such as limited screen size, potential distractions, and variability in device capabilities across different users. It is essential to carefully consider these factors when designing smartphone-based ABMT interventions to ensure optimal effectiveness and user engagement.

#### The Impact of ABMT Characteristics on Anxiety and Depression Symptoms

Our research findings shed light on the impact of ABMT design characteristics on intervention outcomes for anxiety symptoms, providing valuable insights into the diverse factors that shape the efficacy of ABMT interventions. One pivotal aspect of these design characteristics is the influence of threat stimuli, a factor that significantly affects the effectiveness of ABMT. Our results align with previous studies, such as those conducted by Xia et al [61], emphasizing how the nature of these stimuli notably shapes the efficacy of ABMT interventions. Another critical factor is the spatial arrangement of stimuli, whether presented in a top-down or left-right fashion. Interestingly, the top-down arrangement is found to significantly reduce anxiety symptoms, echoing the findings of a previous meta-analysis that demonstrated the impact of spatial stimulus display on the outcome of ABMT treatment [61]. It is noteworthy that the design style, whether gamified or not, does not significantly impact ABMT outcomes. This finding can be linked to a recent review on gamified ABMT, where 2 (50%) out of 4 studies did not reduce mental health problems, while the other 2 (50%) studies did. These mixed results highlight the need for the further exploration of gamified ABMT, as identified in the review, given the limited number of studies conducted in this field [61].

Moreover, the duration for which stimuli are displayed significantly influences ABMT’s effectiveness, with a 200-millisecond display duration emerging as a significant moderator compared to a 500-millisecond display duration. This indicates that shorter exposure durations may lead to more substantial reductions in anxiety symptoms. This aligns with prior research, such as the work by Charles et al [63], emphasizing the importance of optimizing stimulus presentation duration in ABMT protocols. In addition, a co-design study by Zhang et al [64], involving both health care professionals and patients, aimed to enhance conventional ABMT. Their recommendation to initiate training with a lengthier stimulus presentation interval and then gradually reduce the interval has proven instrumental in enhancing engagement and reducing assessment time. Subsequently, Zhang et al [65] adopted this approach, reinforcing the effectiveness of a 200-millisecond duration by presenting participants with a 500-millisecond fixation cross, followed by images for 200 milliseconds.

From the ABMT protocol perspective, the risk of bias does not appear to impact ABMT outcomes, as evidenced by nonsignificant trends in both low and some concern categories. This corresponds with an existing meta-analysis study that has shown the role of bias risk and intervention types in determining the outcomes of ABMT interventions [27]. Finally, the findings from this study on the effect of intervention types (active and placebo) on ABMT outcomes reveal that the intervention types in the anxiety treatment group do not significantly impact ABMT outcomes. In summary, these significant moderators offer nuanced insights into optimizing the design and implementation of ABMT interventions for anxiety, establishing direct connections to existing literature and enhancing the understanding of the multifaceted influences on treatment effectiveness.

When considering threat stimuli, there is a nonsignificant negative effect for both face and words, suggesting a subtle reduction in depression symptoms. The stimulus array type shows no significant impact for either left-right or top-down arrangement. The design style, whether gamified or not, does not significantly influence ABMT outcomes for depression. Notably, within the depression treatment group, both active and placebo interventions exhibit negative but nonsignificant effects, indicating comparable impacts on ABMT outcomes. These findings provide a detailed understanding of the role of these moderators in shaping the effectiveness of ABMT interventions for depression.

### Limitations

This meta-analysis provides valuable insights into the effectiveness of active and placebo ABMT interventions for reducing mental health problems, particularly anxiety and depression. However, it is crucial to acknowledge several limitations that should be considered when interpreting these findings. First, the study’s reliance on small sample sizes within the selected studies limits the generalizability of the results. Future research with larger and more diverse samples could provide a more comprehensive understanding of the effects of ABMT on mental health problems, as current research has primarily focused on high-income countries. Second, the high heterogeneity observed among the included study samples poses a challenge to drawing definitive conclusions. This heterogeneity calls for further investigation into which specific elements of ABMT are the most impactful in reducing mental health problems and whether certain subgroups of individuals may benefit more than others. Despite the significant heterogeneity observed among the study samples, sensitivity analysis revealed that the meta-analysis maintained its statistical significance. The sensitivity analysis conducted in this study should be interpreted with caution. While it helps assess the robustness of the findings, it relies on assumptions that may not always hold. The role of moderators in influencing the effectiveness of ABMT interventions deserves further attention. This meta-analysis highlights certain moderators, such as the type of threat stimuli and intervention duration, but the complex interplay of these factors requires more in-depth investigation to determine their precise impact on treatment outcomes. The availability of internet facilities could be considered an obstacle when contemplating smartphone-delivered ABMT, as it might limit access for certain patients. Last, most of the studies focused on symptoms of mental health conditions and not a formal diagnosis of mental health conditions; further research is needed to validate the use of smartphone-delivered ABMT in this patient collective.

### Future Directions

The findings of this meta-analysis point toward several promising avenues for the future of ABMT research. First, there is a need for the further exploration of design styles in ABMT interventions, with a particular focus on creating engaging and gamified programs that enhance user engagement and motivation. Second, the personalization of ABMT based on individual characteristics and preferences holds significant potential, enabling tailored interventions that match specific symptom profiles and cognitive processes. The standardization of ABMT protocols, including stimulus types, array formats, trial numbers, and intervention durations, is crucial to address the heterogeneity among study samples. Long-term follow-up studies are essential to assess the durability of ABMT’s effects and its potential for preventing symptom relapse. Overall, the future of ABMT research should prioritize enhancing design styles, embracing personalization, addressing heterogeneity, and investigating long-term effects to maximize the effectiveness of ABMT in reducing mental health problems.

### Conclusions

This systematic review and meta-analysis have shed light on the effectiveness of ABMT in addressing mental health symptoms. The findings reveal that active ABMT shows promise in reducing attentional biases, with a moderate overall effect size. However, its impact on directly alleviating anxiety and depression symptoms appears limited, as indicated by smaller and nonsignificant effect sizes within these subgroups. Interestingly, the placebo ABMT results emphasize the influence of belief and expectation in treatment outcomes, highlighting the importance of rigorous study designs to distinguish genuine effects from placebos. Moreover, moderator variables, such as the choice of threat stimuli, design style, and stimulus array type, emerge as critical factors influencing treatment efficacy, underscoring the need for personalized interventions. These findings provide valuable insights for tailoring and optimizing ABMT interventions for individuals with mental health problems.

## Appendix

Multimedia Appendix 1 PRISMA (Preferred Reporting Items for Systematic Review and Meta-Analyses) guidelines.

Multimedia Appendix 2 Risk of bias.

Multimedia Appendix 3 Sensitivity analysis.

Multimedia Appendix 4 Funnel plots.

Multimedia Appendix 5 Moderator analysis.

Multimedia Appendix 6 Raw data.

## Abbreviations

ABMT: attentional bias modification training
CBT: cognitive behavioral therapy
PRISMA: Preferred Reporting Items for Systematic Review and Meta-Analyses
PTSD: posttraumatic stress disorder
