# Supplementary material for: Smartphone-Delivered Attentional Bias Modification Training for Mental Health: Systematic Review and Meta-Analysis
Source: JMIR Ment Health. 2024 Sep 2;11:e56326. doi: 10.2196/56326 (PMC11406109; doi:10.2196/56326)
Supplement: Multimedia Appendix 2 [file mental_v11i1e56326_app2.docx]

| *Study* | ***Risk of bias*** | | | | |  |
| --- | --- | --- | --- | --- | --- | --- |
|  | *Randomization* | *Deviation from intended intervention* | *Missing outcome data* | *Measurement of the outcome* | *Selection of the reported result* | *Overall*  *Score* |
| Enock et al, 2014 [42] | Low | Low | Low | Low | Low | **Low** |
| Yang et al, 2017 [44] | Low | Low | Unclear | Low | Unclear | Some concerns |
| Flaudias et al, 2022 [49] | Low | Low | Low | Low | High | *Low* |
| Dennis-Tiwary et al, 2016[43] | Low | Low | Low | Low | Low | *Low* |
| Teng et al, 2019 [45] | Low | Low | Low | Low | Low | *Low* |
| Dennis et al, 2014 [41] | Low | Low | Low | Low | Low | *Low* |
| Charvet et al, 2021 [23] | Some concerns | Some concerns | Low | Low | Low | *Low* |
| Niles et al, 2020 [47] | Low | Low | Low | Low | Low | *Low* |
| Flaudias et al, 2020 [46] | Low | Low | Low | Low | Low | *Low* |
| Dennis-Tiwary et al, 2017 [24] | Low | Low | Low | Low | Low | *Low* |
| Robinson et al, 2022 [50] | Low | Low | Low | Low | Low | *Low* |
