# Supplementary material for: Smartphone-Delivered Attentional Bias Modification Training for Mental Health: Systematic Review and Meta-Analysis
Source: JMIR Ment Health. 2024 Sep 2;11:e56326. doi: 10.2196/56326 (PMC11406109; doi:10.2196/56326)
Supplement: Multimedia Appendix 3 [file mental_v11i1e56326_app3.docx]

1. Active ABMT for Reducing Mental Health Problems g(p-value) = --0.1777(0.0284)

| Study Removed | Symptoms | Effect Size | p-Value |
| --- | --- | --- | --- |
| Charvet et al, 2021 [23] | Anxiety | -0.181 | 0.0345 |
| Charvet et al, 2021 [23] | Depression | -0.182 | 0.028 |
| Dennis et al, 2014 [41] short | Anxiety | -0.186 | 0.028 |
| Dennis et al, 2014 [41], long | Anxiety | -0.184 | 0.030 |
| Enock et al, 2014 [42] | Anxiety | -0.153 | 0.060 |
| Enock et al, 2014 [42] | Depression | -0.163 | 0.052 |
| Dennis-Tiwary et al, 2016 [43] | Anxiety | -0.190 | 0.022 |
| Dennis-Tiwary et al, 2017 [24] | Anxiety | -0.193 | 0.020 |
| Dennis-Tiwary et al, 2017 [24] | Stress | -0.191 | 0.023 |
| Dennis-Tiwary et al, 2017 [24] | Depression | -0.192 | 0.021 |
| Teng et al, 2019 [45] | Depression | -0.183 | 0.031 |
| Teng et al, 2019 [45] | Anxiety | -0.183 | 0.033 |
| Yang et al, 2017 [44] | Anxiety | -0.182 | 0.028 |
| Flaudias et al, 2020 [46] | Alcohol | -0.166 | 0.050 |
| Robinson et al, 2022 [43] | Anxiety | -0.217 | 0.004 |
| Robinson et al, 2022 [43] | Substance | -0.128 | 0.059 |
| Niles et al, 2020 [47]-NP | Anxiety | -0.184 | 0.031 |
| Niles et al, 2020 [47]-NP | PTSD | -0.156 | 0.061 |
| Niles et al, 2020 [47]-P | Anxiety | -0.178 | 0.038 |
| Niles et al, 2020 [47]-P | PTSD | -0.158 | 0.059 |

1. Placebo ABMT for Reducing Mental Health Problems g(p-value) = -0.3811 (0.0077)

| Study | Symptoms | Effect Size | p-Value |
| --- | --- | --- | --- |
| Dennis et al, 2014 [41], short | Anxiety | -0.280 | 0.009 |
| Dennis et al, 2014 [41], long | Anxiety | -0.412 | 0.006 |
| Enock et al, 2014 [42] | Anxiety | -0.369 | 0.010 |
| Enock et al, 2014 [42] | Depression | -0.382 | 0.006 |
| Dennis-Tiwary et al, 2016 [43] | Anxiety | -0.390 | 0.004 |
| Dennis-Tiwary et al, 2017 [24] | Anxiety | -0.411 | 0.015 |
| Dennis-Tiwary et al, 2017 [24] | Stress | -0.409 | 0.016 |
| Dennis-Tiwary et al, 2017 [24] | Depression | -0.410 | 0.011 |
| Teng et al, 2019 [45] | Depression | -0.159 | 0.013 |
| Teng et al, 2019 [45] | Anxiety | -0.195 | 0.000 |
| Robinson et al, 2022 [50] | Anxiety | -0.177 | 0.006 |
| Robinson et al, 2022 [50] | Substance | -0.189 | 0.001 |
| Niles et al, 2020 [47] | PTSD | -0.179 | 0.004 |
| Niles et al, 2020 [47] | Anxiety | -0.164 | 0.010 |

1. Active Bias g(p-value) = -0.1676(0.0041)

| Study | Study | Effect Size | p-Value |
| --- | --- | --- | --- |
| Dennis et al, 2014 [41], short | Anxiety | -0.189 | 0.001 |
| Dennis et al, 2014 [41], long | Anxiety | -0.179 | 0.004 |
| Enock et al, 2014 [42] | Anxiety | -0.164 | 0.010 |
| Dennis-Tiwary et al, 2016 [43] | Anxiety | -0.169 | 0.006 |
| Dennis-Tiwary et al, 2017 [24] | Anxiety | -0.176 | 0.004 |
| Teng et al, 2019 [45] | Anxiety | -0.134 | 0.015 |
| Yang et al, 2017 [44] | Anxiety | -0.136 | 0.016 |
| Flaudias et al, 2020 [46] | Substance | -0.160 | 0.011 |
| Flaudias et al, 2022 [49] | Anxiety | -0.159 | 0.013 |
| Robinson et al, 2022 [50] | Substance | -0.195 | 0.000 |
| Robinson et al, 2022 [50] | Anxiety | -0.177 | 0.006 |

1. Placebo Bias g(p-value) = -0.0369(0.6582)

| Study Removed | Symptoms | Effect Size | p-Value |
| --- | --- | --- | --- |
| Dennis et al, 2014 [41], short | Anxiety | -0.028 | 0.772 |
| Dennis et al, 2014 [41], long | Anxiety | -0.054 | 0.563 |
| Enock et al, 2014 [42] | Anxiety | -0.024 | 0.803 |
| Dennis-Tiwary 2016 | Anxiety | -0.035 | 0.687 |
| Dennis-Tiwary 2017 | Anxiety | -0.048 | 0.602 |
| Teng et al, 2019 [45] | Anxiety | -0.056 | 0.279 |
| Robinson et al, 2022 [50] | Substance | -0.066 | 0.473 |
| Robinson et al, 2022 [50] | Anxiety | -0.076 | 0.397 |
