# Supplementary material for: Smartphone-Delivered Attentional Bias Modification Training for Mental Health: Systematic Review and Meta-Analysis
Source: JMIR Ment Health. 2024 Sep 2;11:e56326. doi: 10.2196/56326 (PMC11406109; doi:10.2196/56326)

1. Active ABMT for Mental Health Problems


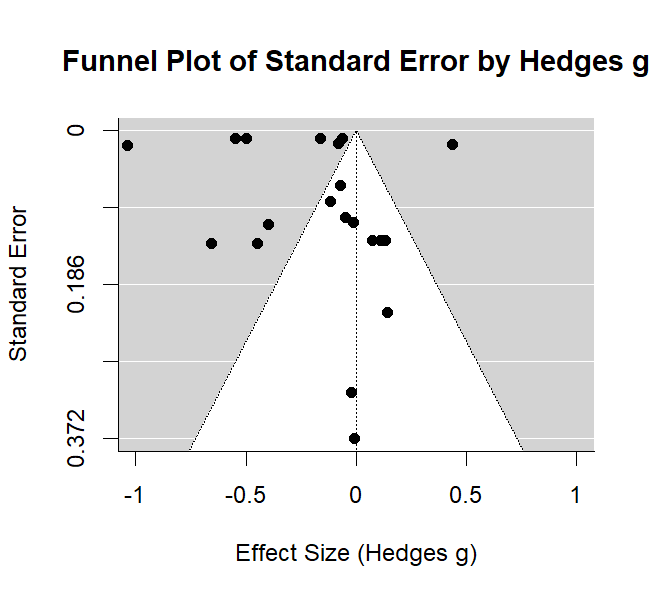


1. Placebo ABMT for Menta Health Problems


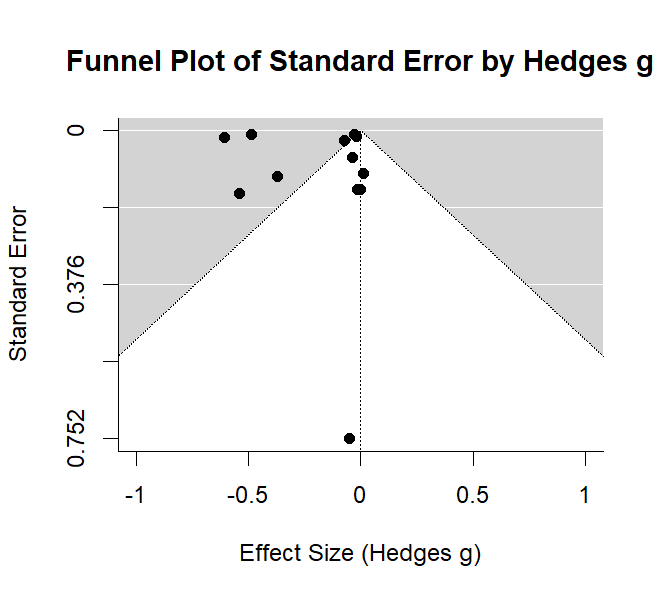


1. Active ABMT for Attention Bias


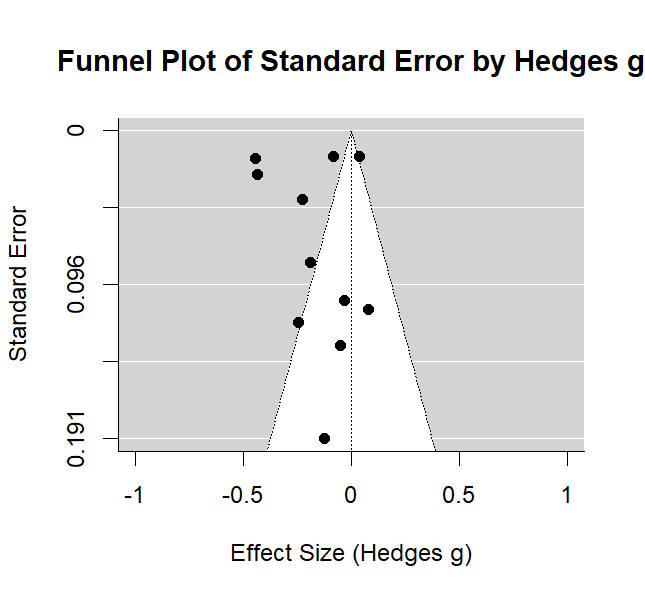


1. Placebo ABMT for Attention Bias


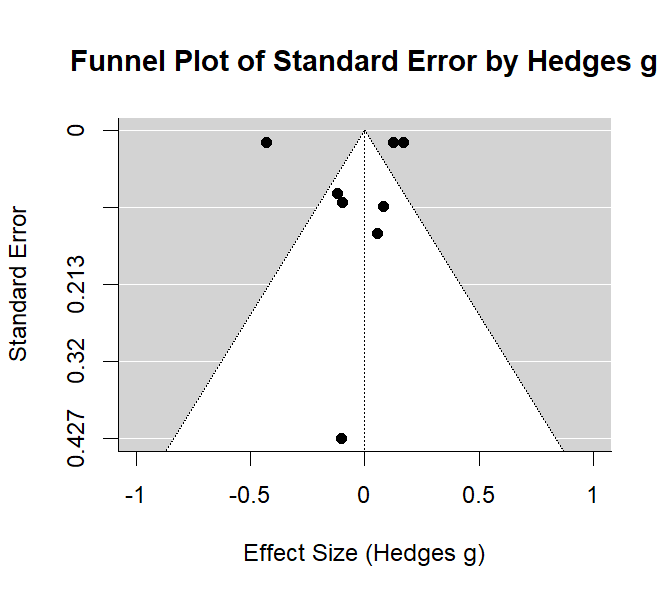

Supplement: Multimedia Appendix 4 [file mental_v11i1e56326_app4.docx]
