# Supplementary material for: Smartphone-Delivered Attentional Bias Modification Training for Mental Health: Systematic Review and Meta-Analysis
Source: JMIR Ment Health. 2024 Sep 2;11:e56326. doi: 10.2196/56326 (PMC11406109; doi:10.2196/56326)
Supplement: Multimedia Appendix 5 [file mental_v11i1e56326_app5.docx]

1. Moderators of ABMT^a^ intervention effectiveness on anxiety symptoms using meta-regression analyses.

|  |  |  |  | 95% CI | |  |  |  |
| --- | --- | --- | --- | --- | --- | --- | --- | --- |
| Moderator | N | Beta | SE | Lower | Upper | Z Value | Q | P value |
|  |  |  |  |  |  |  |  |  |
| **Threat Stimuli** |  |  |  |  |  |  |  |  |
| Face | 11 | -0.100 | 0.084 | -0.265 | 0.065 | -1.187 | 73.632 | 0.235 |
| Images | 11 | 0.536 | 0.189 | 0.165 | 0.907 | 2.830 | 73.632 | 0.005 |
| Words | 11 | -0.001 | 0.129 | -0.254 | 0.252 | -0.009 | 73.632 | 0.993 |
| **Stimulus Array Type**^b^ |  |  |  |  |  |  |  |  |
| Left-Right | 11 | 0.178 | 0.160 | -0.136 | 0.491 | 1.109 | 570.879 | 0.267 |
| Top-Down | 11 | -0.279 | 0.182 | -0.635 | 0.078 | -1.531 | 570.879 | 0.126 |
| **Design Style** |  |  |  |  |  |  |  |  |
| Gamified | 11 | 0.008 | 0.131 | -0.248 | 0.265 | 0.062 | 1007.088 | 0.951 |
| Not Gamified | 11 | -0.084 | 0.174 | -0.425 | 0.257 | -0.482 | 1007.088 | 0.630 |
| **Display Duration** |  |  |  |  |  |  |  |  |
| 200ms | 11 | 0.436 | 0.140 | 0.161 | 0.711 | 3.105 | 73.637 | 0.002 |
| 500ms | 11 | -0.537 | 0.151 | -0.833 | -0.242 | -3.562 | 73.637 | 0.000 |
| **Risk of Bias** |  |  |  |  |  |  |  |  |
| Low | 11 | -0.040 | 0.086 | -0.209 | 0.129 | -0.464 | 1007.096 | 0.643 |
| Some Concerns | 11 | 0.019 | 0.415 | -0.794 | 0.831 | 0.045 | 1007.096 | 0.964 |
| **Anxiety Treatment Group** |  |  |  |  |  |  |  |  |
| Active | 16 | -0.029 | 0.174 | -0.371 | 0.311 | -0.171 | 1809.79 | 0.864 |
| Placebo | 16 | -0.340 | 0.243 | -0.817 | 0.135 | -1.404 | 1809.79 | 0.160 |
| **Attention Bias Treatment Group** |  |  |  |  |  |  |  |  |
| Active | 14 | -0.195 | 0.085 | -0.363 | -0.028 | -2.289 | 604.48 | 0.022 |
| Placebo | 14 | 0.120 | 0.123 | -0.120 | 0.36010563 | 0.9751163 | 604.48 | 0.329 |

^a^ABMT: attention bias modification training

1. Moderators of ABMT intervention effectiveness on depression symptoms using meta-regression analyses.

|  |  |  |  | 95% CI | |  |  |  |
| --- | --- | --- | --- | --- | --- | --- | --- | --- |
| Moderator | N | Beta | SE | Lower | Upper | Z Value | Q | P value |
|  |  |  |  |  |  |  |  |  |
| **Threat Stimuli** |  |  |  |  |  |  |  |  |
| Face | 4 | -0.138 | 0.084 | -0.265 | 0.065 | -1.187 | 73.632 | 0.235 |
| Words | 4 | 0.066 | 0.129 | -0.254 | 0.252 | -0.009 | 73.632 | 0.993 |
| **Stimulus Array Type**^a^ |  |  |  |  |  |  |  |  |
| Left-Right | 4 | -0.072 | 0.160 | -0.136 | 0.491 | 1.109 | 570.879 | 0.267 |
| Top-Down | 4 | -0.066 | 0.182 | -0.635 | 0.078 | -1.531 | 570.879 | 0.126 |
| **Design Style** |  |  |  |  |  |  |  |  |
| Not Gamified | 4 | 0.082 | 0.131 | -0.248 | 0.265 | 0.062 | 1007.088 | 0.951 |
| Gamified | 4 | -0.317 | 0.174 | -0.425 | 0.257 | -0.482 | 1007.088 | 0.630 |
| **Depression Treatment Group** |  |  |  |  |  |  |  |  |
| Active | 7 | -0.119 | 0.122 | -0.359 | 0.121 | -0.972 | 16.08 | 0.331 |
| Placebo | 7 | -0.023 | 0.176 | -0.368 | 0.322 | -0.129 | 16.08 | 0.897 |

^a^ABMT: attention bias modification training
