# Supplementary material for: Smartphone-Delivered Attentional Bias Modification Training for Mental Health: Systematic Review and Meta-Analysis
Source: JMIR Ment Health. 2024 Sep 2;11:e56326. doi: 10.2196/56326 (PMC11406109; doi:10.2196/56326)
Supplement: Multimedia Appendix 6 [file mental_v11i1e56326_app6.docx]

1. Active ABMT for Mental Health Symptom Reduction

| Study | Effect Size | SE | Symptoms | Tasks | Threat stimuli | Number of Stimuli | Stimulus Array Type | Design Style | Display Duration (MS) | Trials |
| --- | --- | --- | --- | --- | --- | --- | --- | --- | --- | --- |
| Charvet et al, 2021 [23] | -0.1169 | 0.086 | Anxiety | Dot Probe | Face | 2 | Top-Down | Gamified | 500 | 120 |
| Charvet et al, 2021 [23] | -0.0075 | 0.372 | Depression | Dot Probe | Face | 2 | Top-Down | Gamified | 500 | 120 |
| Dennis et al, 2014 [41], short | -0.011 | 0.1111 | Anxiety | Dot Probe | Face | 2 | Top-Down | Gamified | 500 | 480 |
| Dennis et al, 2014 [41], long | -0.048 | 0.1053 | Anxiety | Dot Probe | Face | 2 | Top-Down | Gamified | 500 | 640 |
| Enock et al, 2014 [42] | -0.658 | 0.137 | Anxiety | Dot Probe | Face | 2 | Top-Down | Not-Gamified | 500 | 160 |
| Enock et al, 2014 [42] | -0.45 | 0.136 | Depression | Dot Probe | Face | 3 | Top-Down | Not-Gamified | 500 | 160 |
| Dennis-Tiwary et al, 2016 [43] | 0.141 | 0.22 | Anxiety | Dot Probe | Face | 2 | Top-Down | Gamified | 500 | 480 |
| Dennis-Tiwary et al, 2017 [24] | 0.1334 | 0.1334 | Anxiety | Dot Probe | Face | 2 | Top-Down | Gamified | 500 | 160 |
| Dennis-Tiwary et al, 2017 [24] | 0.0752 | 0.1334 | Stress | Dot Probe | Face | 2 | Top-Down | Gamified | 500 | 160 |
| Dennis-Tiwary et al, 2017 [24] | 0.1109 | 0.1334 | Depression | Dot Probe | Face | 2 | Top-Down | Gamified | 500 | 160 |
| Teng et al, 2019 [45] | -0.0718 | 0.0667 | Depression | Dot Probe | Word | 2 | Left-Right | Not-Gamified | 500 | 144 |
| Teng et al, 2019 [45] | -0.081 | 0.0162 | Anxiety | Dot Probe | Word | 2 | Left-Right | Not-Gamified | 500 | 144 |
| Yang 2017 | -0.0214 | 0.316 | Anxiety | Dot Probe | Face | 2 | Top-Down | Not-Gamified | 500 | 800 |
| Flaudias 2020 | -0.3964 | 0.1133 | Alcohol | Stroop | Images | 3 | Grid | Not-Gamified | 3000 | 240 |
| Robinson et al, 2022 [50] | 0.4361 | 0.0165 | Anxiety | Dot Probe | Images | 2 | Left-Right | Not-Gamified | 200 | 440 |
| Robinson et al, 2022 [50] | -1.0388 | 0.0183 | Substance | Stroop | word | 1 | Center | Not-Gamified | 3000 | 160 |
| Niles et al, 2020 [47] | -0.0606 | 0.0102 | Anxiety | Dot Probe | Word | 2 | Top-Down | Not-Gamified | 500 | 70 |
| Niles et al, 2020 [47] | -0.5481 | 0.0103 | PTSD | Dot Probe | Word | 2 | Top-Down | Not-Gamified | 500 | 70 |
| Niles et al, 2020 [47] | -0.1615 | 0.0102 | Anxiety | Dot Probe | Word | 2 | Top-Down | Not-Gamified | 500 | 70 |
| Niles et al, 2020 [47] | -0.4993 | 0.0102 | PTSD | Dot Probe | Word | 2 | Top-Down | Not-Gamified | 500 | 70 |

NP: Non personalized, P: Personalized

1. Placebo ABMT for Mental Health Problems

| Study | Effect Size | SE | Symptoms | Tasks | Threat stimuli | Number Of Stimuli | Stimulus Array Type | Design Style | Display Duration (MS) | Trials |
| --- | --- | --- | --- | --- | --- | --- | --- | --- | --- | --- |
| Dennis et al, 2014 [41], short | -1.7315 | 0.1375 | Anxiety | Dot Probe | Face | 2 | Top-Down | Gamified | 500 | 480 |
| Dennis et al, 2014 [41], long | 0.0102 | 0.1053 | Anxiety | Dot Probe | Face | 2 | Top-Down | Gamified | 500 | 640 |
| Enock et al, 2014 [42] | -0.5396 | 0.1528 | Anxiety | Dot Probe | Face | 2 | Top-Down | Not-Gamified | 500 | 160 |
| Enock et al, 2014 [42] | -0.37 | 0.112 | Depression | Dot Probe | Face | 3 | Top-Down | Not-Gamified | 500 | 160 |
| Dennis-Tiwary et al, 2016 [43] | -0.0502 | 0.7516 | Anxiety | Dot Probe | Face | 2 | Top-Down | Gamified | 500 | 480 |
| Dennis-Tiwary et al, 2017 [24] | -0.0212 | 0.0142 | Anxiety | Dot Probe | Face | 2 | Top-Down | Gamified | 500 | 160 |
| Dennis-Tiwary et al, 2017 [24] | -0.0125 | 0.1429 | Stress | Dot Probe | Face | 2 | Top-Down | Gamified | 500 | 160 |
| Dennis-Tiwary et al, 2017 [24] | -0.0018 | 0.1429 | Depression | Dot Probe | Face | 2 | Top-Down | Gamified | 500 | 160 |
| Teng et al, 2019 [45] | -0.0744 | 0.0257 | Depression | Dot Probe | Word | 2 | Left-Right | Not-Gamified | 500 | 144 |
| Teng et al, 2019 [45] | -0.039 | 0.0667 | Anxiety | Dot Probe | Word | 2 | Left-Right | Not-Gamified | 500 | 144 |
| Robinson et al, 2022 [50] | -0.6086 | 0.017 | Anxiety | Dot Probe | Images | 2 | Left-Right | Not-Gamified | 200 | 440 |
| Robinson et al, 2022 [50] | -1.1821 | 0.0193 | Substance | Stroop | word | 1 | Center | Not-Gamified | 3000 | 160 |
| Niles et al, 2020 [47] | -0.4872 | 0.0102 | PTSD | Dot Probe | Word | 2 | Top-Down | Not-Gamified | 500 | 70 |
| Niles et al, 2020 [47] | -0.0297 | 0.0099 | Anxiety | Dot Probe | Word | 2 | Top-Down | Not-Gamified | 500 | 70 |

1. Active ABMT for Reducing Attention Bias

| Study | Effect Size | SE | Symptoms | Tasks | Threat stimuli | Number Of Stimuli | Stimulus Array Type | Design Style | Display Duration (MS) | Trials |
| --- | --- | --- | --- | --- | --- | --- | --- | --- | --- | --- |
| Dennis et al, 2014 [41], short | 0.081 | 0.1112 | Anxiety | Dot Probe | Face | 2 | Top-Down | Gamified | 500 | 480 |
| Dennis et al, 2014 [41], long | -0.0331 | 0.1053 | Anxiety | Dot Probe | Face | 2 | Top-Down | Gamified | 500 | 640 |
| Enock et al, 2014 [42] | -0.19 | 0.082 | Anxiety | Dot Probe | Face | 2 | Top-Down | Not-Gamified | 500 | 160 |
| Dennis-Tiwary et al, 2016 [43] | -0.1254 | 0.191 | Anxiety | Dot Probe | Face | 2 | Top-Down | Gamified | 500 | 480 |
| Dennis-Tiwary et al, 2017 [24] | -0.0499 | 0.1334 | Anxiety | Dot Probe | Face | 2 | Top-Down | Gamified | 500 | 160 |
| Teng et al, 2019 [45] | -0.4454 | 0.0171 | Anxiety | Dot Probe | Word | 2 | Left-Right | Not-Gamified | 500 | 144 |
| Yang et al, 2017 [44] | -0.4344 | 0.0273 | Anxiety | Dot Probe | Face | 2 | Top-Down | Not-Gamified | 500 | 800 |
| Flaudias et al, 2020 [46] | -0.2427 | 0.119 | Alcohol | Stroop | Images | 3 | Grid | Not-Gamified | 3000 | 240 |
| Flaudias et al, 2022 [49] | -0.2238 | 0.0427 | Alcohol | Stroop | Images | 4 | Grid | Not-Gamified | 500 | 60 |
| Robinson et al, 2022 [50]Stroop | 0.0393 | 0.0161 | Substance | Dot Probe | Images | 2 | Left-Right | Not-Gamified | 200 | 440 |
| Robinson et al, 2022 [50]Dot | -0.0824 | 0.0161 | Substance | Stroop | word | 1 | Center | Not-Gamified | 3000ms | 160 |

1. Placebo ABMT for Reducing Attention Bias

| Study | Effect Size | SE | Symptoms | Tasks | Threat stimuli | Number Of Stimuli | Stimulus Array Type | Design Style | Display Duration (MS) | Trials |
| --- | --- | --- | --- | --- | --- | --- | --- | --- | --- | --- |
| Dennis et al, 2014 [41], short | -0.0982 | 0.1001 | Anxiety | Dot Probe | Face | 2 | Top-Down | Gamified | 500ms | 480 |
| Dennis et al, 2014 [41], long | 0.0824 | 0.1054 | Anxiety | Dot Probe | Face | 2 | Top-Down | Gamified | 500ms | 640 |
| Enock et al, 2014 [42] | -0.12 | 0.087 | Anxiety | Dot Probe | Face | 2 | Top-Down | Not-Gamified | 500ms | 160 |
| Dennis-Tiwary et al, 2016 [43] | -0.1024 | 0.4266 | Anxiety | Dot Probe | Face | 2 | Top-Down | Gamified | 500ms | 480 |
| Dennis-Tiwary et al, 2017 [24] | 0.057 | 0.1429 | Anxiety | Dot Probe | Face | 2 | Top-Down | Gamified | 500ms | 160 |
| Teng et al, 2019 [45] | -0.429 | 0.0171 | Anxiety | Dot Probe | Word | 2 | Left-Right | Not-Gamified | 500MS | 144 |
| Robinson et al, 2022 [50] | 0.1236 | 0.0164 | Anxiety | Dot Probe | Images | 2 | Left-Right | Not-Gamified | 200ms | 440 |
| Robinson et al, 2022 [50] | 0.1687 | 0.0165 | Substance | Stroop | word | 1 | Center | Not-Gamified | 3000ms | 160 |

1. Anxiety treatment group (Active and Placebo)

| Study | Effect Size | SE | Symptoms | Group |
| --- | --- | --- | --- | --- |
| Charvet et al, 2021 [23] | -0.1169 | 0.086 | Anxiety | Active |
| Dennis et al, 2014 [41], short | -0.011 | 0.1111 | Anxiety | Active |
| Dennis et al, 2014 [41], long | -0.048 | 0.1053 | Anxiety | Active |
| Enock et al, 2014 [42] | -0.658 | 0.137 | Anxiety | Active |
| Dennis-Tiwary et al, 2016 [43] | 0.141 | 0.22 | Anxiety | Active |
| Dennis-Tiwary et al, 2017 [24] | 0.0442 | 0.0094 | Anxiety | Active |
| Teng et al, 2019 [45] | -0.099 | 0.0145 | Anxiety | Active |
| Yang et al, 2017 [44] | -0.0214 | 0.316 | Anxiety | Active |
| Robinson et al, 2022 [50] | 0.4361 | 0.0165 | Anxiety | Active |
| Niles et al, 2020 [47]-NP | -0.0606 | 0.0102 | Anxiety | Active |
| Niles et al, 2020 [47]-P | -0.1615 | 0.0102 | Anxiety | Active |
| Dennis et al, 2014 [41], short | 0.081 | 0.1112 | Anxiety | Placebo |
| Dennis et al, 2014 [41], long | -0.0331 | 0.1053 | Anxiety | Placebo |
| Enock et al, 2014 [42] | -0.19 | 0.082 | Anxiety | Placebo |
| Dennis-Tiwary et al, 2016 [43] | -0.1254 | 0.191 | Anxiety | Placebo |
| Dennis-Tiwary et al, 2017 [24] | -0.0499 | 0.1334 | Anxiety | Placebo |
| Teng et al, 2019 [45] | -0.4454 | 0.0171 | Anxiety | Placebo |
| Yang et al, 2017 [44] | -0.4344 | 0.0273 | Anxiety | Placebo |

NP: Non personalized, P: Personalized

1. Attention Bias Treatment Group (Active and Placebo)

| Study | Effect Size | SE | Symptoms (Anxiety) | Group |
| --- | --- | --- | --- | --- |
| Dennis et al, 2014 [41], short | 0.081 | 0.1112 | Attention bias | Active |
| Dennis et al, 2014 [41], long | -0.0331 | 0.1053 | Attention bias | Active |
| Enock et al, 2014 [42] | -0.19 | 0.082 | Attention bias | Active |
| Dennis-Tiwary et al, 2016 [43] | -0.1254 | 0.191 | Attention bias | Active |
| Dennis-Tiwary et al, 2017 [24] | -0.0499 | 0.1334 | Attention bias | Active |
| Teng et al, 2019 [45] | -0.4454 | 0.0171 | Attention bias | Active |
| Yang et al, 2017 [44] | -0.4344 | 0.0273 | Attention bias | Active |
| Dennis et al, 2014 [41], short | -0.0982 | 0.1001 | Attention bias | Placebo |
| Dennis et al, 2014 [41], long | 0.0824 | 0.1054 | Attention bias | Placebo |
| Enock et al, 2014 [42] | -0.12 | 0.087 | Attention bias | Placebo |
| Dennis-Tiwary et al, 2016 [43] | -0.1024 | 0.426615 | Attention bias | Placebo |
| Dennis-Tiwary et al, 2017 [24] | 0.057 | 0.1429 | Attention bias | Placebo |
| Teng et al, 2019 [45] | -0.429 | 0.0171 | Attention bias | Placebo |
| Robinson et al, 2022 [50] | 0.1236 | 0.0164 | Attention bias | Placebo |

1. Depression Treatment Group (Active and Placebo)

| Study | Effect Size | SE | Symptoms (Anxiety) | Group |
| --- | --- | --- | --- | --- |
| Charvet et al, 2021 [23] | -0.0075 | 0.372 | Depression | Active |
| Enock et al, 2014 [42] | -0.45 | 0.136 | Depression | Active |
| Dennis-Tiwary et al, 2017 [24] | 0.1109 | 0.1334 | Depression | Active |
| Teng et al, 2019 [45] | -0.0718 | 0.0667 | Depression | Active |
| Enock et al, 2014 [42] | -0.37 | 0.112 | Depression | Placebo |
| Dennis-Tiwary et al, 2017 [24] | -0.0018 | 0.1429 | Depression | Placebo |
| Teng et al, 2019 [45] | -0.0516 | 0.0667 | Depression | Placebo |
